# Supplementary material for: Mechanisms governing the pioneering and redistribution capabilities of the non-classical pioneer PU.1
Source: Nat Commun. 2020 Jan 21;11:402. doi: 10.1038/s41467-019-13960-2 (PMC6972792; doi:10.1038/s41467-019-13960-2)
Supplement: Supplementary file 7 — Source data [file 41467_2019_13960_MOESM7_ESM.zip › Source_Data/FigureS2/FigureS2B_MotifScanOutput/KmeansCluster3/homerResults/motif30.similar.html]

motif30

## Information for motif30

A
G
T
C
C
G
A
T
A
G
T
C
T
C
G
A
A
G
T
C
A
G
C
T
C
T
G
A
A
G
C
T
G
T
A
C
C
G
T
A
A
G
T
C
T
C
A
G
C
T
G
A
T
A
C
G
  
Reverse Opposite:  

A
G
T
C
G
A
C
T
A
G
T
C
T
C
A
G
A
C
G
T
A
C
T
G
T
C
G
A
A
G
C
T
C
T
G
A
C
T
A
G
A
C
G
T
C
T
A
G
C
G
T
A
C
T
A
G
  

|  |  |
| --- | --- |
| p-value: | 1e-34 |
| log p-value: | -7.854e+01 |
| Information Content per bp: | 1.790 |
| Number of Target Sequences with motif | 85.0 |
| Percentage of Target Sequences with motif | 1.78% |
| Number of Background Sequences with motif | 105.1 |
| Percentage of Background Sequences with motif | 0.25% |
| Average Position of motif in Targets | 104.7 +/- 48.9bp |
| Average Position of motif in Background | 102.9 +/- 55.6bp |
| Strand Bias (log2 ratio + to - strand density) | 0.7 |
| Multiplicity (# of sites on avg that occur together) | 1.00 |
| Motif File: | file (matrix) reverse opposite |

### Similar de novo motifs found

|  |  |  |  |  |  |  |  |
| --- | --- | --- | --- | --- | --- | --- | --- |
| Rank | Match Score | Redundant Motif | P-value | log P-value | % of Targets | % of Background | Motif file |
| 1 | 0.783 | G A T C C T G A G A T C C G A T A G T C C G T A A G T C C G A T C T G A G C A T G A T C T C G A | 1e-29 | -68.184842 | 1.28% | 0.13% | motif file (matrix) |
| 2 | 0.797 | G T A C C G T A G A T C G C A T T A G C G T C A A G T C A G C T C G T A G A C T G A T C G C T A A G T C | 1e-24 | -56.646714 | 1.07% | 0.11% | motif file (matrix) |
